# Traumatic costovertebral joint dislocation

**DOI:** 10.1136/bcr-2020-234931

**Published:** 2020-08-18

**Authors:** Anna H M Mennen, Kristian J de Ruiter, Daphne van Embden

**Affiliations:** Trauma Surgery, Amsterdam UMC, Amsterdam, The Netherlands

**Keywords:** orthopaedic and trauma surgery, orthopaedics, trauma

## Abstract

Rib fractures due to blunt trauma are a common chest injury seen at the emergency department; however, injuries to the costovertebral joints are very rare. We present a case of a 24-year-old man who was admitted after a high-speed car collision and was assessed in a level 1 trauma centre in Amsterdam. He had multiple injuries, including dislocation of the costovertebral joint of ribs 7–10. After performing a literature search we concluded that patients with traumatic costovertebral joint dislocations have a high incidence of vertebral fractures, neurological deficits and additional fractures. We believe that isolated dislocation of one or multiple costovertebral joint(s) can safely be treated conservatively. Close monitoring of the patients is advisable as these injuries are caused by high impact and are associated with other injuries.

## Background

Rib fractures due to blunt trauma are a common chest injury seen at the emergency department^1^; however, injuries to the costovertebral joints are very rare. This injury has been described in association with child abuse,^2 3^ but there is limited known about traumatic costovertebral dislocations in adults.

The costovertebral joints and its ligaments are involved in both respiratory function and thoracic spine stability, but its anatomy is relatively unknown and therefore frequently overlooked during the clinical practice.

All 12 ribs articulate to the vertebral column with two gliding type synovial joints; the costocentral and the costotransverse joint. The costocentral joint is located between the head of the rib and the lateral portion of the vertebral centrum, and the costotransverse is located between the tubercle of the rib and the tip of the transverse process. In recent literature the term ‘costovertebral’ is interchangeably used to describe either the costocentral or the costotransverse joint, or both. In this case report we will refer to the combination of these two joints when describing the costovertebral articulation. There are multiple ligaments that attach a rib to the vertebrae on most levels; however, the 1st, 10th, 11th and 12th rib have no intra-articular ligament. Furthermore, the first rib also has no superior costotransverse ligament.^4^ Because of its position at the top of the rib cage and the lack of stabilising ligaments, the first rib is more prone to fracture or dislocation, just as the 10th, 11th and 12th.^5^ Due to its close relation to the spinal cord thorough assessment and imaging to rule out neurological damage is strongly advised when treating a patient with a costovertebral dislocation.

To the best of our knowledge, no similar case involving a dislocation of the costovertebral joint that dislocated to anterior has been described in the literature yet.

## Case presentation

A 24-year-old man was admitted after a high-speed car collision into a highway guardrail. The car was severely damaged and all airbags were out. Immediately after the collision he climbed out of his car by himself and started to walk around seemingly distraught and not adequately responding to questions from the paramedics.

He was assessed in a level 1 trauma centre in Amsterdam according the Advanced Trauma Life Support (ATLS) principles. During the primary survey there were no signs of airway problems and his C-spine was immobilised. He had no respiratory problems and with oxygen suppletion through a non-rebreathing mask he had a saturation of 99%. He had no clinical signs of a (tension)pneumothorax or haemothorax. On the left side of this chest a seat belt sign was seen. His pulse was 114/min with a blood pressure of 98/60 mm Hg. There were some excoriations on his abdomen, but when palpated it was non-rigid and not painful. There was no suspicion of a pelvic or femur fracture. He was sedated because of agitation due to possible head injury or drug or alcohol intoxication. Pupils were equal and reactive to light. Furthermore, during the log-roll and spinal palpation no haematoma or other signs of fractures were seen and our now lightly sedated patient did not have symptoms of pain and wounds were not seen.

During secondary survey a small head wound on the back of the head was seen, as well as multiple excoriations on the thorax, abdomen, pelvic area and right knee. A Morell-Lavallee lesion on the right hip was also noted. Because of the vertebral fractures and possible head injury a neurologist was consulted to perform a neurological examination at the trauma room during the secondary survey. The neurological examination took place after 5 mg midazolam was intravenously given because the patient was exhibiting motor restlessness. No major neurological deficit was found during the examination. It was noted that the patient moved his arms and legs spontaneously and that the plantar reflex was normal on both sides.

## Investigations

**Figure 1:**
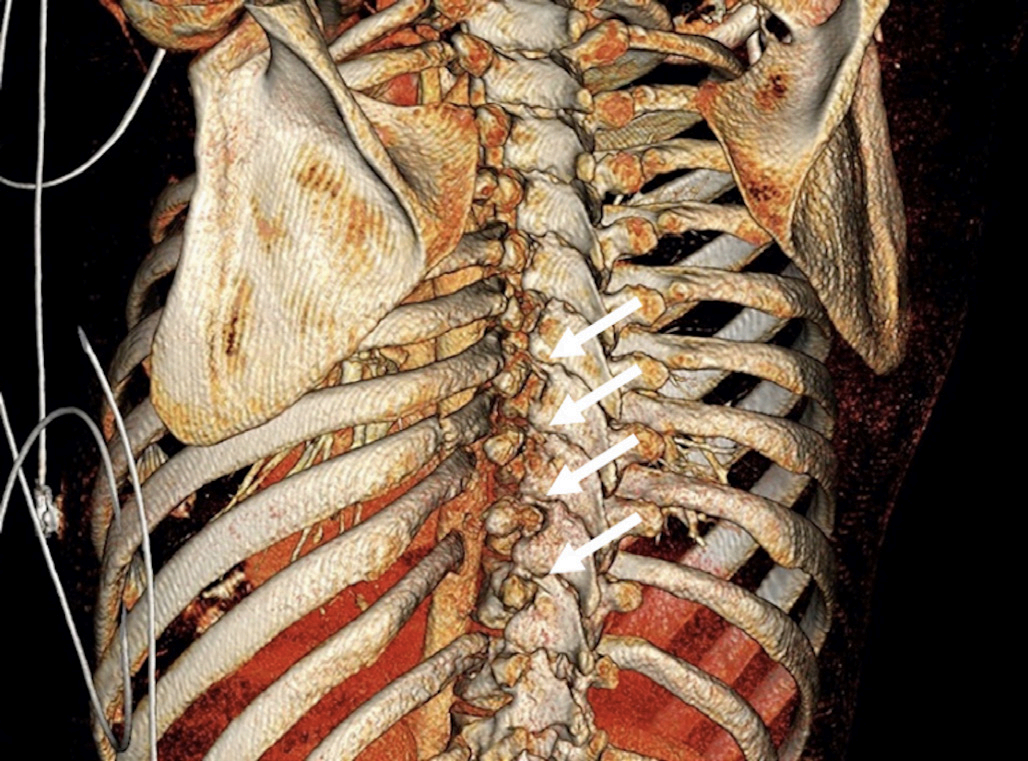
Coronal three-dimensional CT-scan. The arrows show the costovertebral dislocation of rib 7–10 on the left side. Fractures of the left rib 3–8, 11 and 12 can also be seen.

**Figure 2:**
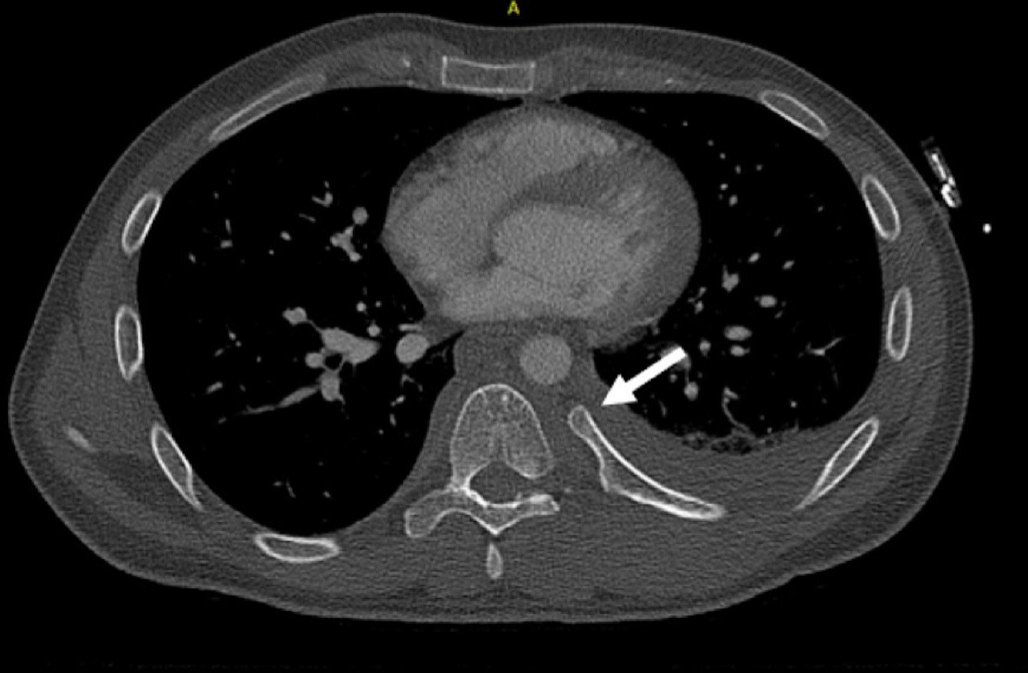
Transverse CT-scan made during the initial trauma assessment. The arrow shows the costovertebral dislocation of the left seventh rib.

During the initial trauma assessment, a total body CT was performed, which showed the following extensive injuries:

- A paravertebral blush located at the left ribs 9 and 10 intercostal arteries.
- A flail chest with haemopneumothorax on the left side, and a pneumothorax and pulmonary contusion on the right side.
- A non-displaced sternal fracture.
- Fractures of ribs 3–8, 11 and 12 on the left side; all posterior and most of them displaced and closely related to the costovertebral joint (figure 1).
- Dislocation of the costovertebral joint of ribs 7–10 on the left side (figures 1 and 2).
- Transverse process fractures of the thoracic spine at level 5–9 (Th5-9) on the left side and lumbar spine level 2–3 bilateral (L2-3).
- A L4 and spinal process fracture with a lateral wedge causing displacement in the coronal plane (AO classification AO_A1-N0-M1^6^).

Due to fact that the patient kept exhibiting motor restlessness a brain CT was repeated 1 day after the initial trauma assessment which showed no intracranial bleeding or fractures of the skull.

## Treatment

The patient was successfully treated with a chest drain on the left side of his chest. He also underwent an angiography and a successful coiling of the paravertebral blush. The following day the lumbar fracture was stabilised using spinal fusion. The rib fractures and costovertebral rib dislocations were treated non-operatively and the pain was managed with opioid analgesia.

## Outcome and follow-up

After being admitted to the surgical ward for 7 days, the patient was discharged. The patient visited the outpatient clinic 2 and 6 weeks after discharge and showed little symptoms of pain. He did appear to have a winged scapula on the left side. The winged scapula improved spontaneously so it was possibly caused by compression from a local haematoma on the long thoracic nerve or accessory nerve or by the haematoma itself. After 3 months, the electromyography did not show any nerve injury and the clinical signs of the winged scapula were improving. He regained a full range of motion of the left shoulder.

## Discussion

As described earlier, the anatomy of the costovertebral joint consists of two joints named the costocentral and costotransverse joint and several ligaments. For this joint to dislocate either concomitant rib fractures have to occur or ligamentous injury. The majority of posterior rib fractures occur at the rib neck due to the strong costovertebral ligamentous attachment to the rib head and tubercle,^4^ this kind of stress fracture is also well described as a rowers and swimmers injury.^7 8^ The costovertebral ligaments are innervated by the lateral branch of the thoracic dorsal rami of C8 and Th1-Th11, and some studies suggest that these ligaments add a protective mechanism against traction and compression of the nerves by maintaining proper positioning of the nerves in the intervertebral foramen.^9^ Since the ligaments often get injured during the dislocation of the costovertebral joints, it is not surprising that neurological damage would occur in a patient with a costovertebral dislocation. There are multiple causes for scapular winging, including iatrogenic, idiopathic and traumatic injuries. While injury of the long thoracic nerve resulting in paralysis of the serratus anterior is the most common traumatic cause,^10^ direct traumatic injury to the insertion of the serratus anterior or scapular fractures have also been described to cause scapular winging.^11^ If neurological deficits are found, further work-up is necessary to determine the cause and possible treatment of it.

Despite the fact that most dislocations, such as sternoclavicular joint dislocations are relocated,^12^ we feel that an isolated dislocation of the costovertebral articulation can safely be managed conservative. We support our statement that costovertebral dislocation can be safely treated conservatively with findings from a literature search we performed on 3 January 2019 using the electronic databases of Pubmed, the Cochrane library and EMBASE. The keywords and MeSH terms Costovertebral AND injury OR fracture OR dislocation OR subluxation were used which resulted in a total of 42 articles of which 3 described cases of costovertebral dislocation in adults with a traumatic aetiology.^5 13 14^

A total of eight cases of costovertebral dislocation were described in these three articles. The most common mechanism of injury was a motor vehicle collision (n=7). The lower costovertebral joints were most often involved, although two cases of a first costovertebral joint dislocation were described. Almost all cases had vertebral fractures accompanying the dislocation (n=7), one fracture was located at T1 and the rest is located at the lower thoracic or lumbar vertebrae. Neurological deficits were apparent in five cases, paraplegia was the most common one (n=4), all of these cases were suffering from vertebral fractures. In six out of eight cases there were additional fractures, namely; five cases with rib fractures, two scapula fractures, one sternum fracture, one femur fracture and one acromioclavicular joint dislocation. Four cases had associated injuries, with intrathoracic injuries (n=5) and intra-abdominal injuries (n=4) being the most common ones. Two patients died because of their sustained injuries.

Although the costovertebral dislocation itself seldom leads to complications and the mortality that was noted was never due to the dislocation, these types of injuries are a significant clinical sign of very high impact trauma and there should be a high suspicion on severe other spinal, chest or abdominal injuries.

Especially, one should be aware for aortic injury, considering the anatomic proximity of the dislocated rib to the aorta. While it is a very rare complication, some cases of traumatic aortic injury have been described in combination with displaced posterior rib fractures in other case reports.^15–19^ Some of these aortic injuries occurred after several days, while during the initial trauma assessment there was no sign of vascular damage. Close monitoring during admission is therefore strongly advised, and at the first signs of haemodynamic instability or increased chest tube drainage this kind of complication should be considered. While rib fractures in the left side of the chest are currently not an indication for surgical stabilisation, in recent literature costotransverse screw placement is described as a feasible technique for management of paraspinal rib fractures to prevent the development of aortic injury.^20^

**Learning points**

- Patients with traumatic costovertebral joint dislocations have a high incidence of vertebral fractures, neurological deficits and additional fractures.
- We believe that isolated dislocation of one or multiple costovertebral joint(s) can safely be treated conservatively.
- Close monitoring of the patients is advisable as these injuries are caused by high impact and are associated with other injuries.
